# Altered resting-state functional connectivity patterns in late middle-aged and older adults with obstructive sleep apnea

**DOI:** 10.3389/fneur.2023.1215882

**Published:** 2023-06-19

**Authors:** Guillermo Martinez Villar, Véronique Daneault, Marie-Ève Martineau-Dussault, Andrée-Ann Baril, Katia Gagnon, Chantal Lafond, Danielle Gilbert, Cynthia Thompson, Nicola Andrea Marchi, Jean-Marc Lina, Jacques Montplaisir, Julie Carrier, Nadia Gosselin, Claire André

**Affiliations:** ^1^Center for Advanced Research in Sleep Medicine, Hôpital du Sacré-Coeur de Montréal, Centre Intégré Universitaire de Santé et de Services Sociaux du Nord de l’Île-de-Montréal, Montréal, QC, Canada; ^2^Department of Psychology, Université de Montréal, Montréal, QC, Canada; ^3^Douglas Mental Health Institute, McGill University, Montréal, QC, Canada; ^4^Laboratory and Sleep Clinic, Hôpital en Santé Mentale Rivière-des-Prairies, Centre Intégré Universitaire de Santé et de Services Sociaux du Nord de l’Île-de-Montréal, Montréal, QC, Canada; ^5^Department of Pulmonology, Hôpital du Sacré-Coeur de Montréal, Centre Intégré Universitaire de Santé et de Services Sociaux du Nord de l’Île-de-Montréal, Montréal, QC, Canada; ^6^Department of Medicine, Université de Montréal, Montreal, QC, Canada; ^7^Department of Radiology, Radio-Oncology and Nuclear Medicine, Université de Montréal, QC, Canada; ^8^Department of Radiology, Hopital du Sacré-Coeur de Montréal, CIUSSS du Nord-de-l’Ile-de, Montréal, QC, Canada; ^9^Center for Investigation and Research in Sleep, Department of Medicine, Lausanne University Hospital and University of Lausanne, Lausanne, Switzerland; ^10^Laboratory for Research in Neuroimaging, Department of Clinical Neurosciences, Lausanne University Hospital and University of Lausanne, Lausanne, Switzerland; ^11^Département de Génie Electrique, École de Technologie Supérieure, Montréal, QC, Canada; ^12^Department of Psychiatry, Université de Montréal, Montréal, QC, Canada

**Keywords:** sleep-disordered breathing, aging, resting-state functional connectivity, default mode network, medial temporal lobe, hippocampus, medial prefrontal cortex, posterior cingulate cortex

## Abstract

**Introduction:**

Obstructive sleep apnea (OSA) is increasingly recognized as a risk factor for cognitive decline, and has been associated with structural brain alterations in regions relevant to memory processes and Alzheimer’s disease. However, it is unclear whether OSA is associated with disrupted functional connectivity (FC) patterns between these regions in late middle-aged and older populations. Thus, we characterized the associations between OSA severity and resting-state FC between the default mode network (DMN) and medial temporal lobe (MTL) regions. Second, we explored whether significant FC changes differed depending on cognitive status and were associated with cognitive performance.

**Methods:**

Ninety-four participants [24 women, 65.7 ± 6.9 years old, 41% with Mild Cognitive Impairment (MCI)] underwent a polysomnography, a comprehensive neuropsychological assessment and a resting-state functional magnetic resonance imaging (MRI). General linear models were conducted between OSA severity markers (i.e., the apnea-hypopnea, oxygen desaturation and microarousal indices) and FC values between DMN and MTL regions using CONN toolbox. Partial correlations were then performed between OSA-related FC patterns and (i) OSA severity markers in subgroups stratified by cognitive status (i.e., cognitively unimpaired versus MCI) and (ii) cognitive scores in the whole sample. All analyzes were controlled for age, sex and education, and considered significant at a *p* < 0.05 threshold corrected for false discovery rate.

**Results:**

In the whole sample, a higher apnea-hypopnea index was significantly associated with lower FC between (i) the medial prefrontal cortex and bilateral hippocampi, and (ii) the left hippocampus and both the posterior cingulate cortex and precuneus. FC patterns were not associated with the oxygen desaturation index, or micro-arousal index. When stratifying the sample according to cognitive status, all associations remained significant in cognitively unimpaired individuals but not in the MCI group. No significant associations were observed between cognition and OSA severity or OSA-related FC patterns.

**Discussion:**

OSA severity was associated with patterns of lower FC in regions relevant to memory processes and Alzheimer’s disease. Since no associations were found with cognitive performance, these FC changes could precede detectable cognitive deficits. Whether these FC patterns predict future cognitive decline over the long-term needs to be investigated.

## 1. Introduction

Obstructive sleep apnea (OSA) is a sleep-related breathing disorder characterized by the repetitive narrowing of the pharyngeal airway, resulting in nocturnal episodes of complete (apnea) or partial (hypopnea) obstruction (1). OSA prevalence in adults ranges from 6 to 17% but increases with aging, affecting up to 49% of the elderly population (2). By chronically fragmenting sleep and provoking intermittent episodes of hypoxemia, OSA may increase oxidative stress, neuroinflammation and cerebral edema, leading to complex and potentially non-linear changes in neuroimaging markers such as cerebral gray matter volume, metabolism, white matter diffusivity, as well as greater amyloid and tau levels (3–10). Unsurprisingly, most epidemiological studies show that untreated OSA increases the risk of cognitive decline and dementia (11–15).

Structural neuroimaging has been used to identify OSA-related brain changes suggestive of early neurodegenerative processes in late middle-aged and older adults. Some brain regions appear to be particularly sensitive to the pathological consequences of OSA, such as the medial temporal lobe (MTL), and other hubs of the default mode network (DMN), such as the posterior cingulate cortex and the precuneus (4, 5, 7, 9, 10). Whether OSA is associated with functional connectivity (FC) abnormalities in older populations is unclear, as most studies have been performed in young and middle-aged adults, often in male participants only. Studies among 20–70 years old participants with moderate to severe OSA show that they have lower resting-state FC between the hippocampus and other DMN regions, such as the posterior cingular cortex (PCC), medial prefrontal cortex (mPFC) and parahippocampal region (PHC) compared to non-apneic participants (16, 17). They also have lower FC between the hippocampus and other brain regions such as the insula, anterior cingulate cortex, thalamus, medial and superior temporal cortex (17, 18). FC increases have also been documented in association with OSA between the MTL and fronto-temporal areas (18, 19), the mPFC and parietal and MTL regions (15, 16, 19, 20), and the posterior insula and both the precuneus and anterior insula (21, 22).

MTL regions are among the earliest cortical regions to degenerate in Alzheimer’s disease (AD) (23, 24), and hub regions of the DMN are prone to accumulate amyloid pathology (25–27). In the context where OSA is increasingly recognized as a risk factor for AD, it is crucial to determine whether OSA is associated with disrupted FC patterns between these regions, especially in late middle-aged and older populations. To date, the only study which was specifically performed in this age group included 72 participants aged 66.6 ± 7.9 years old, at high risk for dementia (i.e., presenting subjective and/or objective cognitive impairments and seeking assessment for cognitive decline). The authors found that higher hypoxemia levels were associated with lower FC between the left and right parahippocampal gyri in participants with OSA, as compared to those without OSA (28). Therefore, these results suggest that MTL regions may be sensitive to OSA. It is therefore of interest to confirm and extend these findings to a cohort including both cognitively unimpaired and individuals with mild cognitive impairment (MCI) to better understand the effects of OSA on FC patterns in aging.

The present study aimed at characterizing the association between OSA severity and resting-state FC between regions of the DMN and MTL in late middle-aged and older adults (from 55 to 90 years old). We explored these associations according to cognitive status (i.e., in those with and without MCI). Moreover, we investigated the associations between FC and episodic memory performance. We hypothesized that more severe OSA would be associated with lower FC between MTL regions (i.e., the hippocampus and parahippocampal cortex) and DMN regions (i.e., the mPFC, lateral parietal cortex, precuneus and PCC). Furthermore, we expected these FC changes to be associated with lower episodic memory performance or the presence of MCI.

## 2. Materials and methods

### 2.1. Participants

Participants were recruited through newspaper advertising and waiting lists from the *Hôpital du Sacré-Coeur de Montréal* sleep apnea clinic. Participants interested by the study were first contacted by phone and screened using a structured interview. All participants included in the present study were aged over 55 years old, fluent in French or English, had a minimum of 7 years of education and presented preserved autonomy in daily life.

They were excluded if they presented any of the following criteria: (1) presence or history of major neurological (e.g., Parkinson’s disease, traumatic brain injury, stroke, epilepsy), psychiatric (e.g., diagnosed anxiety disorder, depression, schizophrenia), or pulmonary disorders (e.g., asthma, chronic obstructive pulmonary disease), (2) dementia diagnosis or suspicion, (3) diabetes; (4) uncontrolled hypertension (>160/100 mmHg); (5) substance abuse; (6) use of medication susceptible to affect brain function, vigilance or sleep; (7) self-reported or PSG-confirmed sleep disorders other than OSA (e.g., insomnia, periodic limb movement disorder, rapid-eye movement sleep behavior disorder, central sleep apnea, etc.), (8) having started an OSA treatment in the past or being currently treated for OSA; (9) body mass index (BMI) > 40 kg/m^2^, (10) contraindications for magnetic resonance imaging (MRI).

Participants were excluded from the analyzes if one of the exclusion criteria was observed after the screening interview, throughout the study testing (e.g., sleep disorder detected at the time of the polysomnography (PSG) recording, anomalies on MRI preventing valid analyzes). Of note, all subjects with OSA were newly diagnosed and untreated and no participant started OSA treatment before the end of the examinations (i.e., between the PSG, neuropsychological and MRI testing).

Written consent was obtained from each participant and the research protocol was approved by the Ethics committees of the *Centre intégré universitaire de santé et de services sociaux du Nord-de-l’île-de-Montréal* and the Functional Neuroimaging Unit of the *Institut universitaire de gériatrie de Montréal* (#2012–697, #12–13-008 and #2010–468).

### 2.2. Protocol overview

Participants were invited for one full night of in-laboratory PSG followed by a comprehensive neuropsychological assessment the next morning. Weight and height were measured at the sleep lab to calculate BMI and participants filled out questionnaires. Participants underwent an MRI scan within a mean interval of 2.4 ± 2.2 months following their PSG recording.

### 2.3. Demographic data and questionnaires

Participants completed a custom questionnaire collecting multiple demographic variables including age, sex and years of education. They also filled several questionnaires [for an exhaustive list, see (4, 10)], including the Epworth Sleepiness Scale (29), the Beck Depression Inventory-II (30), and the Beck Anxiety Inventory (31), respectively assessing daytime sleepiness, depression and anxiety symptoms.

### 2.4. Neuropsychological assessment

Each participant underwent a comprehensive neuropsychological assessment encompassing global cognitive functioning, attention and processing speed, executive functions, language and episodic memory, as previously described (10). MCI diagnoses were established when participants had (1) at least two z-scores below −1.5 standard deviations compared to normative data in one cognitive domain, or (2) a Montreal Cognitive Assessment score < 26 and at least two z-scores below −1.5 standard deviations in multiple cognitive domains (10). All diagnoses were reviewed by a senior neuropsychologist (N.G.). In statistical analyzes, we used immediate and delayed free recall scores at the Rey Auditory Verbal Learning Test [RAVLT] (32) as measures of verbal episodic memory performance.

### 2.5. Polysomnographic recordings

The PSG protocol was fully described in previous articles (4, 33). Briefly, participants underwent one night of in-laboratory PSG recording with 18 electroencephalographic electrodes referred to linked earlobes. We also used thoraco-abdominal strain gages, oronasal canula and thermal sensors, transcutaneous finger pulse oximeter, electrooculograms, chin and anterior tibialis electromyogram, and an electrocardiogram. Sleep and respiratory scoring were performed by an experienced medical electrophysiology technologist based on the American Academy of Sleep Medicine guidelines (34). Sleep onset latency, total sleep time, sleep efficiency, and percentage and duration of each sleep stage were among the variables computed. An apneic episode was defined as a reduction in airflow ≥90% from baseline lasting ≥10 s. An hypopneic episode was defined as a reduction in airflow ≥30% from baseline lasting ≥10 s and accompanied either by an oxygen desaturation ≥3% or by an electroencephalogram arousal. We used three complementary markers of OSA severity: the apnea-hypopnea index (AHI; number of apneas and hypopneas per hour), the oxygen desaturation index (ODI; number of 3% drops in blood oxygenation level per hour) and the micro-arousal index (number of microarousals per hour).

### 2.6. Magnetic resonance imaging acquisition

MRI was acquired using a Magnetom TRIO 3 Tesla scanner (Siemens Healthcare, United States). The sequence included a three-dimensional T1-weighted Turbo Flash multi-echo Magnetization-prepared rapid gradient-echo (MPRAGE) with a 32-channel head coil using the parameters from the E5 Massachusetts General Hospital (Boston, Massachusetts, United States). Acquisition parameters were: repetition time = 2,530 ms/root mean square of 4 echo times = 1.64 ms, 3.50 ms, 5.36 ms, 7.22 ms; matrix size = 256×256; field of view = 256×256 mm; voxel size = 1.0 mm isotropic, flip angle = 7 °; and 176 sagittal orientations. Two resting-state functional MRI time series (150 images each, lasting 6.5 min) were then acquired. The resting-state functional MRI sequences included multislice T2*-weighted fMRI images obtained with a gradient echo-planar sequence (EPI axial) (2D-EP) (42 axial slices; slice thickness 3.4 mm; gap 3.4 mm, matrix size 64 × 64 × 42; repetition time (TR) 2,600 ms; echo time (TE) 30 ms; flip angle 90°, FoV: 218 mm). During the 6.5-min resting-state fMRI sequence, participants were asked to fix a white dot on a black screen. Wakefulness was ensured using a camera. We also acquired T2 and FLAIR sequences, which were not analyzed in the present article, but used to detect clinically-significant anomalies.

### 2.7. Functional MRI images preprocessing

Resting-state fMRI were preprocessed using SPM12 (Wellcome Trust Center for Neuroimaging, London, United Kingdom) and CONN Functional Connectivity SPM Toolbox version 21a^1^ supported by MATLAB R2020b (MatWorks, Inc. Natick, MA, United States). First, structural (T1) and functional images (EPI volumes) from two rs-MRI sessions acquired consecutively (6 min each, 150 volumes) were realigned (with an estimation for the 6 movement parameters) and corrected for differences in time acquisition. Volumes showing a frame displacement greater than 0.5 mm were removed through the Artifact Detection Tools (ART) toolbox implemented in ConnToolbox.^2^ After, co-registration and normalization of data in MNI (Montreal Neurological Institute) stereotaxic space and 8 mm full width at half maximum smoothing was applied. Then, nuisance parameters, such as the effect of movement and physiological artifact, were denoised from the BOLD signal using the “anatomical principal component-based noise correction (aCompCor)” function. Finally, a band-pass filter was applied for frequencies between 0.008 and 0.09 Hz in order to better control for movement. Valid scans from both sessions were merged together prior to connectivity analyzes.

**Figure 1:**
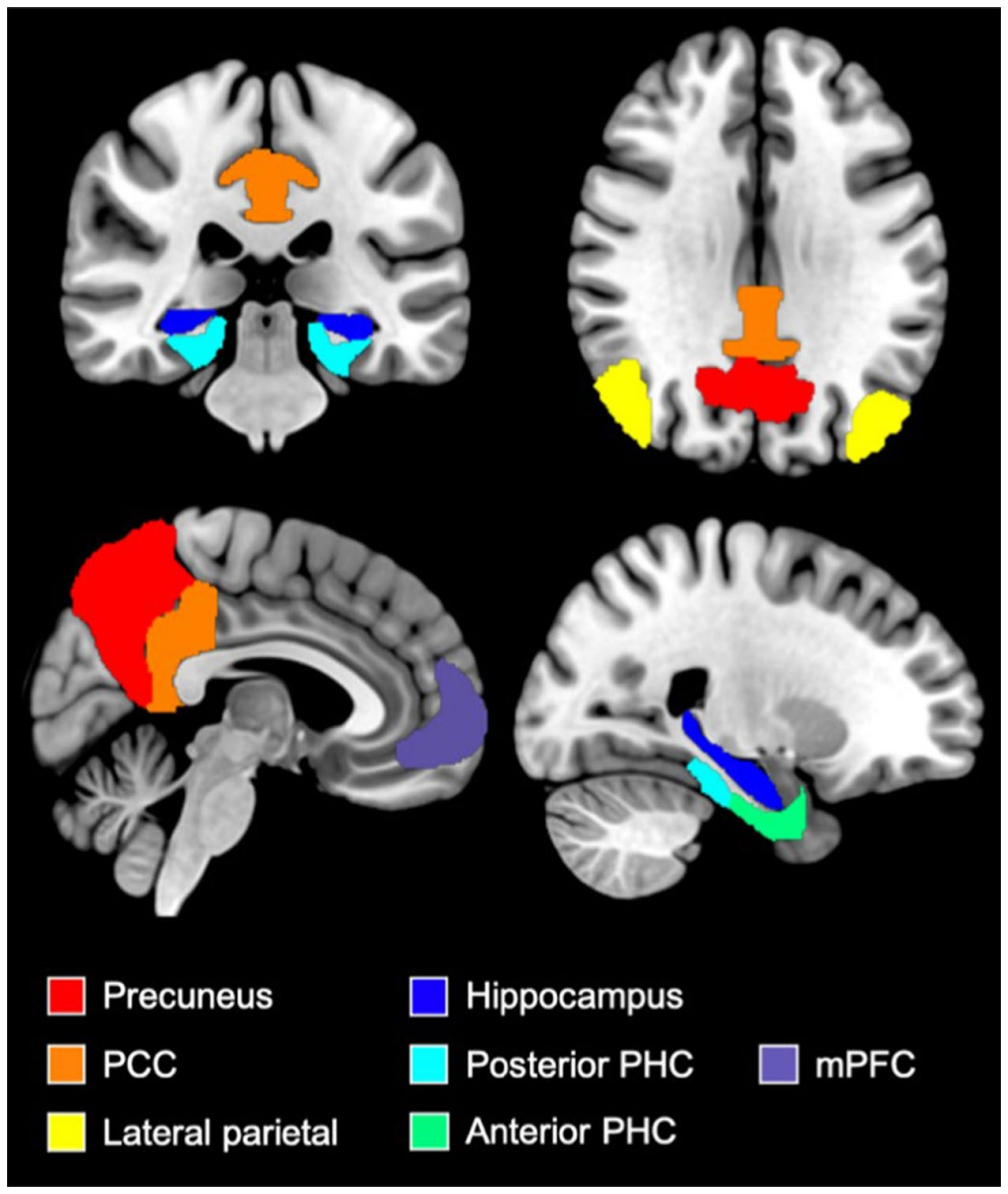
Illustration of ROIs used in FC analyzes. Figure illustrating the medial prefrontal cortex (purple), the precuneus (red), the posterior cingulate cortex (orange), the lateral parietal cortex (yellow), the hippocampi (dark blue), the anterior parahippocampal cortex (green) and the posterior parahippocampal cortex (light blue) ROIs. These ROIs were derived from the DMN network atlas implemented in CONN toolbox (mPFC and LP), Harvard-Oxford atlas (Precuneus, HPC, PHC) and Neuromorphometrics atlas (PCC). mPFC, medial prefrontal cortex; PCC, posterior cingulate cortex; PHC, parahippocampal cortex.

CONN toolbox was used to perform region of interest (ROI)-based connectivity analyzes between 11 regions: the mPFC, left and right lateral parietal cortex (LP), PCC, precuneus, left and right hippocampus (HPC) and left and right anterior and posterior parahippocampal gyri (PHC). The ROIs were derived from the DMN network atlas implemented in CONN toolbox (mPFC and LP), Harvard-Oxford atlas (Precuneus, HPC, PHC) (35–39) and Neuromorphometrics atlas (PCC)^3^ (see Figure 1 for an illustration of selected ROIs).

### 2.8. Statistical analyzes

Demographic, clinical and sleep data were compared between cognitively healthy participants and individuals with MCI using independent samples Student’s *t*-tests for continuous variables and chi-squared tests for categorical variables. All non-normal variables were log-transformed prior to statistical analyzes.

First, we assessed the associations between OSA severity markers (i.e., the AHI, ODI and micro-arousal index) and ROI-to-ROI FC patterns in the whole sample using CONN toolbox. More specifically, first-level analysis aimed at identifying FC patterns between all 11 ROIs used as seeds. This step consisted in computing bivariate Pearson’s correlations between the mean BOLD signal time courses of each pair of ROIs. Resulting correlation coefficients were converted to normally distributed scores using Fisher’s transformation to improve normality assumptions of the subsequent, second-level analyzes. Individual correlation matrices were then entered into a second-level general linear model using data-driven cluster-level inferences based on spatial pairwise clustering (SPC) method (40). T-statistics of the entire ROI-to-ROI matrix were estimated using a general linear model with OSA severity markers as dependent variables, separately, and age, sex and education as covariates. ROIs in this matrix were sorted automatically by a hierarchical clustering procedure (optimal leaf sorting for hierarchical clustering) (18) based on ROI-to-ROI anatomical proximity or functional similarity metrics. Thereafter, this statistical parametric map was thresholded using a significance level of *p* < 0.01 (uncorrected). The resulting suprathreshold areas define a series of non-overlapping clusters. The cluster-level false discovery rate (FDR)-corrected *p* < 0.05 threshold was applied for significance.

Secondly, we aimed at verifying whether (i) the links between OSA severity markers and significant FC changes differed depending on cognitive status, and (ii) FC patterns that were significantly associated with OSA severity in the main analyzes were also correlated with global cognition and memory performance. Therefore, FC values were extracted from the significant clusters obtained above. Partial correlations were performed between FC values and (i) OSA severity markers separately in cognitively unimpaired and MCI individuals, and (ii) cognitive scores in the whole sample. All analyzes were controlled for age, sex and education, and considered significant at a *p* < 0.05 FDR-corrected threshold. Statistical analyzes were conducted using R (version 64 4.1.1) or JASP (version 0.16.1), and the significance threshold was set at *p* < 0.05 after FDR correction for demographical and cognitive analyzes.

## 3. Results

### 3.1. Participants’ characteristics

One-hundred and nine individuals were tested with PSG and MRI following phone screening interview. A total of 15 subjects were excluded for presenting exclusion criteria (e.g., early dementia, asthma, chronic obstructive pulmonary disease, rapid-eye movement sleep behavior disorder, or central sleep apnea; *n* = 6), brain abnormalities detected following MRI inspection by a neuroradiologist (e.g., extensive white matter damage, abnormal levels of atrophy, silent infarcts, meningioma, and large arachnoid cyst; *n* = 6), movement artifacts on resting-state MRI images (*n* = 1), delay >12 months between MRI and PSG examinations (*n* = 1) or outlier regarding respiratory signal data (*n* = 1). Therefore, a total of 94 participants were included in statistical analyzes, whose characteristics are presented in Table 1. Specifically, 57 participants had an AHI below 15 so were considered as controls, and 37 participants had an AHI ≥ 15 so were considered as having moderate-to-severe OSA.

**Table 1:** Participants characteristics in the whole sample and in subgroups stratified by cognitive status.

Variables	Whole sample (*n* = 94)		Cognitively unimpaired (*n* = 55)		MCI (*n* = 39)		Between-groups differences		
	Mean	SD	Mean	SD	Mean	SD	T or χ^2^	df	P_unc._
Demographic and clinical variables									
Age: years	65.8	6.9	66.2	7.0	64.9	6.8	0.91	92	0.37
Sex: nb (%) of women	24 (25.5)		15 (27.3)		9 (23.1)		0.21	1	0.65
BMI: kg/m^2^	26.9	3.3	26.8	3.8	27.1	2.6	−0.42	92	0.67
Education: years	15.3	3.3	15.9	3.1	14.4	3.5	2.15	92	**0.03**
BAI: score	3.6	4.0	3.7	3.9	3.5	4.1	0.27	88	0.79
BDI: score	6.1	5.6	6.0	5.3	6.4	6.0	−0.37	88	0.71
MoCA: total score	27.2	2.3	28.2	1.4	25.8	2.5	5.65	87	**<0.001**
RAVLT IFR: score	9.6	3.3	10.9	2.5	7.7	3.4	5.14	92	**<0.001**
RAVLT DFR: score	9.4	3.4	10.8	2.7	7.3	3.3	5.56	91	**<0.001**
Sleep variables									
Epworth Sleepiness Scale: score	8.2	5.3	8.5	5.7	7.8	4.8	0.61	88	0.54
Insomnia Severity Index: score	6.6	5.3	6.0	5.1	7.3	5.6	−1.13	88	0.26
Sleep onset latency: min	14.2	18.8	11.3	10.5	18.4	26.0	−1.83	92	0.07
Total sleep time: min	357.4	57.4	362.1	57.7	350.9	57.0	0.93	92	0.35
Wake after sleep onset: min	94.1	51.1	93.9	54.6	94.3	46.5	−0.05	92	0.96
Sleep efficiency: %	79.4	10.6	79.6	11.0	79.1	10.0	0.25	92	0.80
NREM-sleep stage 1: % of TST	22.0	12.7	22.7	14.0	21.1	10.5	0.57	92	0.57
NREM-sleep stage 2: % of TST	53.5	10.3	53.2	11.4	53.9	8.6	−0.31	92	0.76
NREM-sleep stage 3: % of TST	8.5	9.3	8.9	10.1	7.8	8.0	0.57	92	0.57
REM sleep: % of TST	16.0	5.5	15.2	5.3	17.1	5.6	−1.73	92	0.09
Apnea-hypopnea index: nb/h	16.5	17.6	17.8	20.3	14.7	13.2	0.85	92	0.40
Oxygen desaturation index: nb/h	8.8	11.0	10.4	11.7	6.7	9.8	1.58	87	0.12
Microarousal index: nb/h	16.6	8.8	17.4	9.9	15.5	7.1	1.05	92	0.30

Participants characteristics are reported as mean ± standard deviation, unless indicated otherwise. Between-group comparisons were assessed with Student’s T-tests for continuous variables and chi-squared tests for categorical variables.

BAI, Beck Anxiety Inventory; BDI, Beck Depression Inventory-II; BMI, body mass index; DFR, delayed free recall; h, hour; IFR, Immediate free recall; MCI, mild cognitive impairment; min, minutes; MoCA, Montreal Cognitive Assessment; nb, number; NREM, non-rapid eye movement; RAVLT, Rey auditory Verbal Learning Test; REM, rapid eye movement; TST, total sleep time; unc, uncorrected.

As expected, cognitively unimpaired and MCI participants differed on several cognitive variables, with MCI participants exhibiting lower global cognition (MoCA score: *t* = −5.65, P_uncorrected_ < 0.001), memory performance (Rey Verbal Episodic Memory Test immediate free recall: *t* = 5.14, P_uncorrected_ < 0.001; delayed free recall: *t* = 5.56, P_uncorrected_ < 0.001). Demographics, sleep parameters and OSA severity variables did not significantly differ between the two groups, except that MCI participants had lower education levels (*t* = 2.15, P_uncorrected_ = 0.03).

### 3.2. Associations between OSA severity markers and functional connectivity in the whole sample

**Figure 2:**
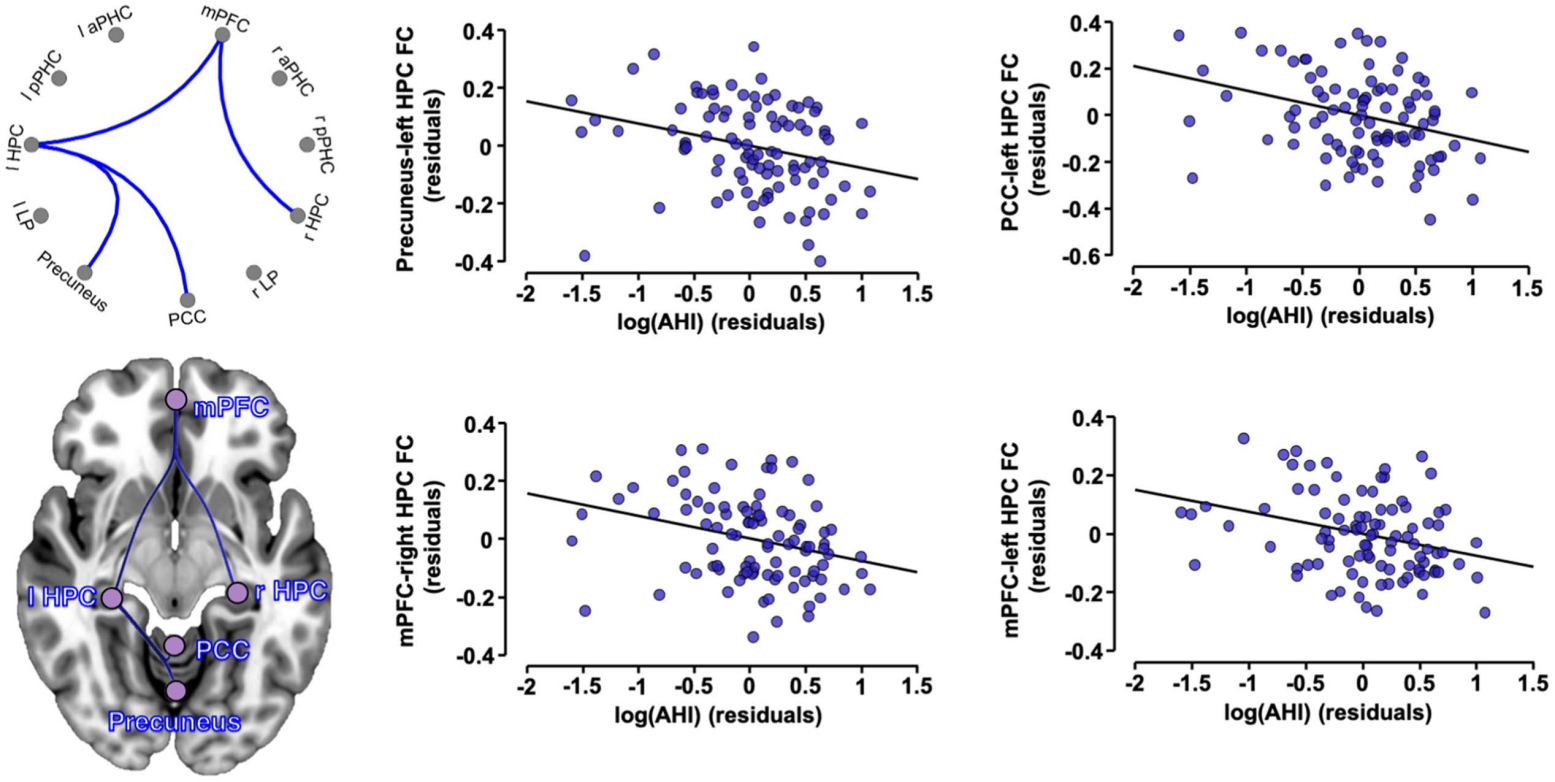
Associations between OSA severity and FC in the whole sample. Partial regression plots between significant ROI-to-ROI FC values (expressed in Fisher Z-scores) and log-transformed apnea-hypopnea index, controlling for age, sex and education. A cluster-level p < 0.05 FDR-corrected threshold was applied for significance. OSA, obstructive sleep apnea; FC, functional connectivity; AHI, apnea-hypopnea index; aPHC, anterior parahippocampal cortex; HPC, hippocampus; l, left; LP, lateral parietal; mPFC, medial prefrontal cortex; PCC, posterior cingulate cortex; pPHC, posterior parahippocampal cortex; r, right.

We first tested the associations between OSA severity markers and FC patterns in the whole sample. Figure 2 shows that higher AHI values are associated with reduced FC among various ROIs, and Table 2 presents a list of significant associations and related statistics. We found that higher AHI was significantly associated with lower FC between (i) the mPFC and both left (*T* = -3.02, *p* = 0.003) and right (*T* = -2.71, *p* = 0.008) hippocampi, and (ii) between the left hippocampus and both the PCC (*T* = -3.27, *p* = 0.002) and precuneus (*T* = -2.90, *p* = 0.005; Figure 2; Table 2). No significant associations were observed between FC patterns and the ODI index or the micro-arousal index.

**Table 2:** Detailed statistics of the significant associations between the AHI and FC values.

FC cluster	Statistics	P_unc._	P_FDR-corrected_
**Cluster 1**	**Mass = 71.12**	**0.006**	**0.006**
PCC–left HPC	T (89) = −3.27	0.002	0.08
mPFC–left HPC	T (89) = −3.02	0.003	0.09
Precuneus–left HPC	T (89) = −2.90	0.005	0.09
mPFC–right HPC	T (89) = −2.71	0.008	0.11

Detailed statistics of significant clusters resulting from multiple regression analyzes between the AHI and resting-state images. Results were considered significant after a cluster-level FDR correction for multiple testing.

AHI, apnea-hypopnea index; FC, functional connectivity; FDR, false discovery rate; HPC, hippocampus; mPFC, medial prefrontal cortex; PCC, posterior cingulate cortex; pPHC, posterior parahippocampal cortex; unc, uncorrected.

As the BMI significantly correlated with the AHI (*r* = 0.34, 95% CI: 0.17–0.48, P_uncorrected_ < 0.001), we verified post-hoc whether controlling for the BMI affected these results. Partial correlations between the AHI and AHI-related FC values extracted from significant clusters in the whole sample remained significant when the BMI was entered as a covariate in the model (Supplementary Table S1).

### 3.3. Impact of cognitive status on the associations between FC and OSA severity

To investigate the impact of cognitive status on the associations between OSA severity markers and FC changes, we performed partial correlations between these variables in subgroups stratified according to cognitive diagnosis (Table 3). For these analyzes, we exclusively used the pairs of ROIs which showed significant FC associations with OSA severity in the whole sample. All associations remained significant in cognitively unimpaired individuals, but not in MCI participants (Table 3).

**Table 3:** Partial correlations between the AHI and AHI-related FC changes according to cognitive status.

AHI-related FC changes		Full sample (*n* = 94)	Cognitively unimpaired (*n* = 55)	MCI (*n* = 39)
Precuneus–left HPC	Pearson’s r	−0.28	−0.28	−0.20
	95% CI	−0.50 – −0.04	−0.49 – −0.02	−0.65 – −0.24
	P_unc._	0.008	0.047	0.23
	P_FDR-corrected_	**0.008**	**0.047**	0.31
PCC - left HPC	Pearson’s r	−0.33	−0.43	−0.17
	95% CI	−0.53 – −0.10	−0.64 – −0.18	−0.53 – −0.21
	P_unc._	0.002	0.001	0.31
	P_FDR-corrected_	**0.006**	**0.004**	0.31
mPFC - left HPC	Pearson’s r	−0.31	−0.33	−0.35
	95% CI	−0.48 – −0.13	−0.53 – −0.10	−0.62 – −0.01
	P_unc._	0.003	0.016	0.038
	P_FDR-corrected_	**0.006**	**0.02**	0.15
mPFC - right HPC	Pearson’s r	−0.29	−0.38	−0.27
	95% CI	−0.48 – −0.09	−0.59 – −0.14	−0.59 – −0.10
	P_unc._	0.005	0.005	0.11
	P_FDR-corrected_	**0.007**	**0.01**	0.22

Partial correlations between the AHI and AHI-related FC values extracted from significant clusters, controlling for age, sex and education. Results in bold are significant after an FDR correction for multiple testing.

AHI, apnea-hypopnea index; FC, functional connectivity; CI, confidence interval; FDR, false discovery rate; HPC, hippocampus; MCI, mild cognitive impairment; mPFC, medial prefrontal cortex; PCC, posterior cingulate cortex; pPHC, posterior parahippocampal cortex; unc, uncorrected.

### 3.4. Associations with global cognition and memory performance

We tested whether significant AHI-related FC patterns described above were associated with global cognition and memory performance in the whole sample. The results are presented in Supplementary Table S2, and no significant association was observed.

## 4. Discussion

This study aimed at characterizing the associations between OSA severity and resting-state FC between brain regions that are sensitive to OSA and AD in late middle-aged and older adults. We found that more severe OSA, as measured with higher AHI, was associated with reduced FC between the hippocampus and the mPFC, the precuneus and PCC. All associations were driven by cognitively unimpaired participants and not replicated in MCI individuals. No association with global cognition or episodic memory performance was observed.

These results show that OSA severity is associated with a pattern of reduced FC between the hippocampus and several other DMN regions, such as the mPFC, PCC and precuneus. Interestingly, DMN regions are known to be structurally affected in relation to OSA. More specifically, animal studies show that the hippocampus is particularly sensitive to hypoxia, which is a major consequence of OSA (41–43). Hippocampal pyramidal neurons are especially vulnerable to hypoxia due to their large size, and their high pre-existing levels of reactive oxygen species (41–43). Consistently, studies in humans show that OSA is associated with hippocampal damage, with greater OSA severity being associated with lower hippocampal volume (9, 44). Interestingly, the MTL is well known to be among the first cortical areas to accumulate tau pathology, a hallmark of AD which partly underlies neurodegeneration in this population (45, 46). Patients with OSA also exhibit increased tau levels (6, 47, 48), which could explain the vulnerability of MTL regions to OSA. In the present study, FC was reduced between the hippocampus and several other DMN regions, namely the mPFC, PCC and precuneus. These DMN hubs are highly metabolically active, and known to be prone to amyloid accumulation in the early stages of the disease (25–27, 49). Previous studies have shown that higher OSA severity is related to structural and metabolic alterations in fronto-parietal areas (5). Moreover, several groups have reported that moderate-to-severe OSA patients exhibit a pattern of increased amyloid deposition in the PCC and precuneus (3, 50). More specifically, a multimodal neuroimaging study has shown that amyloid deposition topographically overlapped with a pattern of increased metabolic activity (3). This suggests that greater levels of neuroinflammation and neuronal hyperactivity could increase amyloid accumulation and precede neurodegeneration in the same brain regions (5, 51). Taken together, these studies suggest that OSA may enhance AD-related pathological mechanisms and neurodegeneration in DMN hubs, which may explain the vulnerability of this network to OSA. Longitudinal studies could bring important new information regarding this hypothesis.

The DMN is also sensitive to sleep disturbances. Indeed, DMN hubs are highly active during wakefulness, but this network is disengaged in deep sleep and exhibits the highest levels of slow wave activity (52–54). However, sleep quality is largely affected by the aging process, with increased fragmentation and wakefulness levels and decreased slow wave sleep (55, 56). Therefore, it is likely that age-related sleep changes may lead to an increase in neuronal activity levels (57, 58), which could in turn enhance amyloid deposition and disrupt brain functioning. Consistently, FC alterations in the DMN were observed by several groups in the context of sleep-deprivation protocols. In young adults, 24 h of sleep deprivation have been associated with a reduction of FC between DMN nodes (59–61). A study showed that the DMN and attentional network are less anti-correlated after sleep deprivation (62).

In older adults, self-reported sleep disturbances, as measured with greater scores on the Pittsburgh Sleep Quality Index, have been associated with disrupted FC in the DMN in patients with MCI (63, 64). Compared to our study, the authors found that the DMN was sensitive to global self-reported sleep disturbances in later cognitive stages (i.e., patients with MCI), but the regions affected included the mPFC, parietal and temporal regions, which parallels our observations. Here, we identify OSA as a condition impacting FC between DMN regions in late middle-aged and older adults. Specifically in older populations at risk for dementia, OSA is associated with FC abnormalities between MTL regions (28). We extend these findings to late-middle aged and older participants who were less at risk of cognitive decline, as 58% of our sample was composed of cognitively unimpaired participants. Interestingly, we found that OSA was associated with more widespread FC changes than those previously observed (28), involving the mPFC, PCC and precuneus. The effect of OSA on FC may be greater in cognitively healthy individuals and less evident in participants more advanced in the AD continuum. Consistent with this hypothesis, analyzes stratified by cognitive status show that in our sample, all associations were significant in cognitively unimpaired participants but not in individuals with MCI. It is possible that after the apparition of the first cognitive signs, the variance in FC explained by OSA severity may be lower than the variance explained by AD pathology.

FC alterations among DMN regions may partially result from microstructural alterations in white matter tracts or gray matter atrophy (65). Several studies show that key DMN nodes are directly connected by white matter tracts (66, 67). FC patterns between the PCC and hippocampus have been associated with the microstructure of white matter tracts connecting these regions, including the cingulum bundle (68). As OSA is known to affect white matter microstructure [for a recent review, see (5)], it is possible that OSA-related FC patterns may be partly explained by the alteration of white matter tracts. However, the mechanisms underlying FC are still unclear for some parts of the DMN, including the mPFC (66, 69, 70), such that the alteration of white matter tracts may not be the only mechanism underlying OSA-related FC changes. The adverse effects of OSA on FC in older adults may also be influenced by gray matter alterations and amyloid deposition, as they have both been associated with FC changes in preclinical and prodromal AD (71–73). In fact, both increases and decreases in FC have been reported in the Alzheimer’s continuum (i.e., from cognitively unimpaired amyloid-positive older adults to MCI patients), probably in reaction to underlying neurodegenerative processes and amyloid deposition (73, 74). In later stages of the disease, FC reductions between DMN regions are more marked (74–76), notably between the hippocampus, the mPFC and PCC in participants with MCI and AD dementia (71, 74, 77, 78). We here show that OSA is associated with disturbed FC patterns between the same regions, especially in cognitively unimpaired individuals. This reinforces the idea that OSA may alter the integrity of the DMN at an early stage (i.e., in participants with likely relatively mild levels of AD pathology), while in more advanced stages of the disease, FC reductions could be more explained by underlying neurodegenerative processes. Further studies including measures of AD biomarkers will need to verify this hypothesis. Of note, the fact that the associations between OSA and FC patterns were driven by cognitively unimpaired individuals in our sample could also be influenced by the lower sample size of the MCI group (i.e., 55 cognitively unimpaired versus 39 MCI participants).

Importantly, the DMN is involved in interoception and episodic memory functioning (79). Lower FC among the DMN and limbic regions has consistently been associated with episodic memory impairment in older adults and patients with MCI or mild AD (71, 74, 80, 81). Moreover, disrupted FC in the DMN precedes the emergence of significant cognitive impairment and cortical atrophy in AD (71). In our sample, we did not observe significant associations between cognitive performance and OSA-related FC alterations. Cognitively unimpaired individuals performed in the normal range for their age and education levels on MoCA and RAVLT scores, such that there was overall little inter-individual performance variability. Therefore, these participants are able to maintain cognitive performance in the normal range despite exhibiting FC-related disruption in relation to OSA. This suggests that OSA may have an early and relatively asymptomatic impact on cerebral connectivity at first, with participants being cognitively resilient to the adverse effects of OSA on cerebral FC. However, OSA may still precipitate cognitive decline assessed with longitudinal designs. Interestingly, OSA may represent a modifiable risk factor for cognitive decline, as some studies suggest that CPAP treatment may improve cognitive functioning in patients with OSA (82–86). However, the results of large multicenter studies are still mixed (87–89). Interestingly, some studies suggest that CPAP treatment may increase FC in adults with OSA, notably in DMN regions such as the PCC and precuneus (82, 90). Taken together, the potential beneficial effects of CPAP treatment to preserve brain and cognitive integrity still need to be confirmed in older populations.

The relatively low proportion of women (i.e., 25.5% of the sample) is a limitation to the present study, preventing from robustly exploring the effect of sex on OSA-related FC disruptions. This question is however important and should be addressed in larger samples. Another limit is the use of one standard neuropsychological test (i.e., the RAVLT) to measure memory, as cognitively unimpaired individuals performed in the normal range for their age and education levels, leading to little statistical variability. A sleep-dependent memory task could have been better suited to detect subtle memory disturbances consequently to OSA in cognitively unimpaired individuals.

## 5. Conclusion

This study shows that OSA is associated with reduced FC between major nodes of the DMN (i.e., MTL, mPFC, PCC and precuneus). As these regions are sensitive to both OSA and AD pathological processes, and crucial for cognition, future studies will need to assess whether OSA-related FC alterations are associated with cognitive decline, and the potential of OSA treatment to maintain the integrity of neural networks.

## Data availability statement

The raw data supporting the conclusions of this article will be made available by the authors, without undue reservation.

## Ethics statement

The studies involving human participants were reviewed and approved by Ethics committees of the Center intégré universitaire de santé et de services sociaux du Nord-de-l’île-de-Montréal and the Functional Neuroimaging Unit of the Institut universitaire de gériatrie de Montréal (#2012–697, #12–13-008 and #2010–468). The patients/participants provided their written informed consent to participate in this study.

## Author contributions

VD, GMV, J-ML, JM, JC, NG, and CA contributed to the design and conceptualization of the study. JM, JC, and NG secured study funding. VD, GMV, M-EM-D, A-AB, KG, CL, DG, CT, NM, and CA contributed to data acquisition and preprocessing. VD and GMV performed statistical analyzes. VD, GMV, CA, and NG interpreted the data and drafted the manuscript. All authors critically revised and approved the final version of the manuscript.

## Funding

This research was supported by the Canadian Institutes of Health Research [Grant numbers FDN154291, MOP123294, and MOP102631] and the Fonds de la Recherche du Québec–Santé. NG holds a Canada Research Chair in sleep disorders and brain health. JM holds a Canada Research Chair in Sleep Medicine. M-EM-D is supported by a doctoral fellowship from the CIHR and the Fonds de Recherche du Québec–Santé. A-AB is supported by the Banting Fellowship Program (CIHR). NM is supported by the Swiss National Science Foundation Postdoctoral Mobility fellowship. CA is supported by postdoctoral fellowships by the Fonds de Recherche du Québec–Nature et Technologies through the Merit scholarship program for foreign students (PBEEE), the Fonds de Recherche du Québec–Santé and Quebec Bio-Imaging network.

## Conflict of interest

JC is the current recipient of an investigator driven grant from Eisai (not linked to the present study). NG has received honoraria from Eisai and sponsorships from Jazz Pharmaceuticals, Eisai and Paladin (not linked to the present study).

The remaining authors declare that the research was conducted in the absence of any commercial or financial relationships that could be construed as a potential conflict of interest.

## Publisher’s note

All claims expressed in this article are solely those of the authors and do not necessarily represent those of their affiliated organizations, or those of the publisher, the editors and the reviewers. Any product that may be evaluated in this article, or claim that may be made by its manufacturer, is not guaranteed or endorsed by the publisher.

## Footnotes

^1^www.conn-toolbox.org

^2^https://www.nitrc.org/projects/artifact_detect/

^3^http://Neuromorphometrics.com/
