# Supplementary material for: Altered resting-state functional connectivity patterns in late middle-aged and older adults with obstructive sleep apnea
Source: Front Neurol. 2023 Jun 19;14:1215882. doi: 10.3389/fneur.2023.1215882 (PMC10353887; doi:10.3389/fneur.2023.1215882)
Supplement: Supplementary file 1 [file Data_Sheet_1.docx]

Supplementary Material

Altered functional MRI connectivity patterns in late middle-aged and older adults with obstructive sleep apnea

**Guillermo Martinez Villar, Véronique Daneault, Marie-Ève Martineau-Dussault, Andrée-Ann Baril, Katia Gagnon, Chantal Lafond, Danielle Gilbert, Cynthia Thompson, Nicola Andrea Marchi, Jean-Marc Lina, Jacques Montplaisir, Julie Carrier, Nadia Gosselin, Claire André**

**Correspondence:** Corresponding Authors: [nadia.gosselin@umontreal.ca](mailto:nadia.gosselin@umontreal.ca) ; [claire.andre@umontreal.ca](mailto:claire.andre@umontreal.ca)

**Supplementary Table 1.** **Partial correlations between OSA severity markers and OSA-related FC changes controlling for age, sex, education and the body mass index.**

| **AHI-related FC changes** | | **3-covariates model** | **4-covariates model** |
| --- | --- | --- | --- |
| Precuneus - left HPC | Pearson's r | -0.28 | -0.29 |
|  | 95% CI | -0.50 – -0.04 | -0.51 – -0.03 |
|  | P_unc._ | 0.008 | 0.006 |
|  | P_FDR-corrected_ | **0.008** | **0.012** |
| PCC - left HPC | Pearson's r | -0.33 | -0.31 |
|  | 95% CI | -0.53 – -0.10 | -0.51 – -0.08 |
|  | P_unc._ | 0.002 | 0.003 |
|  | P_FDR-corrected_ | **0.006** | **0.012** |
| mPFC - left HPC | Pearson's r | -0.31 | -0.24 |
|  | 95% CI | -0.48 – -0.13 | -0.41 – -0.06 |
|  | P_unc._ | 0.003 | 0.021 |
|  | P_FDR-corrected_ | **0.006** | **0.021** |
| mPFC - right HPC | Pearson's r | -0.29 | -0.25 |
|  | 95% CI | -0.48 – -0.09 | -0.45 – -0.03 |
|  | P_unc._ | 0.005 | 0.017 |
|  | P_FDR-corrected_ | **0.007** | **0.021** |

Partial correlations between OSA severity markers (i.e., the AHI and ODI) and OSA-related FC values extracted from significant clusters. The 3-covariates model is controlled for age, sex and education. The 4-covariates model is controlled for age, sex, education and the body mass index. Results in bold are significant after an FDR correction for multiple testing.

*Abbreviations: AHI, apnea-hypopnea index; CI, confidence interval; FDR, false discovery rate; HPC, hippocampus; MCI, mild cognitive impairment; mPFC, medial prefrontal cortex; ODI, oxygen desaturation index; PCC, posterior cingulate cortex; pPHC, posterior parahippocampal cortex.*

**Supplementary Table 2.** **Partial correlations between cognition, the AHI and AHI-related FC patterns in the whole sample.**

| **Variable** |  | **MoCA**  **(n=89)** | **RAVLT immediate free recall (n=94)** | **RAVLT delayed free recall (n=93)** |
| --- | --- | --- | --- | --- |
| log(AHI) | Pearson's r | 0.02 | 0.11 | 0.15 |
|  | 95% CI | -0.20 – 0.26 | -0.09 – 0.30 | -0.05 – 0.36 |
|  | P_unc._ | 0.84 | 0.31 | 0.15 |
|  | P_FDR-corrected_ | 0.99 | 0.5 | 0.375 |
| Precuneus - left HPC | Pearson's r | -0.04 | -0.001 | -0.001 |
|  | 95% CI | -0.28 – 0.19 | -0.19 – 0.17 | -0.21 – 0.20 |
|  | P_unc._ | 0.75 | 0.99 | 0.99 |
|  | P_FDR-corrected_ | 0.99 | 0.99 | 0.99 |
| PCC - left HPC | Pearson's r | -0.09 | -0.09 | -0.08 |
|  | 95% CI | -0.30 – 0.10 | -0.30 – 0.10 | -0.31 – 0.12 |
|  | P_unc._ | 0.40 | 0.40 | 0.44 |
|  | P_FDR-corrected_ | 0.99 | 0.5 | 0.55 |
| mPFC - left HPC | Pearson's r | -0.16 | -0.20 | -0.18 |
|  | 95% CI | -0.38 – 0.08 | -0.37 – -0.05 | -0.36 – -0.02 |
|  | P_unc._ | 0.15 | 0.05 | 0.09 |
|  | P_FDR-corrected_ | 0.75 | 0.25 | 0.375 |
| mPFC - right HPC | Pearson's r | -0.002 | -0.14 | -0.09 |
|  | 95% CI | -0.25 – 0.23 | -0.33 – 0.06 | -0.30 – 0.12 |
|  | P_unc._ | 0.99 | 0.19 | 0.40 |
|  | P_FDR-corrected_ | 0.99 | 0.475 | 0.55 |

Partial correlations between cognitive performance (i.e., global cognition and memory performance) and the AHI and AHI-related FC values extracted from significant clusters, controlling for age, sex and education. No result survived an FDR correction for multiple comparisons.

*Abbreviations: AHI, apnea-hypopnea index; FC: functional connectivity; CI, confidence interval; FDR, false discovery rate; HPC, hippocampus; MCI, mild cognitive impairment; MoCA, Montreal Cognitive Assessment; mPFC, medial prefrontal cortex; PCC, posterior cingulate cortex; pPHC, posterior parahippocampal cortex; RAVLT, Rey Auditory Verbal Learning Test; unc, uncorrected.*
